# An experimental model of contusion injury in humans

**DOI:** 10.1371/journal.pone.0277765

**Published:** 2022-11-17

**Authors:** Matthew J. Barnes, Dominic Lomiwes, David A. D. Parry, Stephen Mackintosh

**Affiliations:** 1 School of Sport, Exercise & Nutrition, Massey University, Palmerston North, New Zealand; 2 The New Zealand Institute for Plant and Food Research Ltd, Palmerston North, New Zealand; 3 School of Natural Sciences, Massey University, Palmerston North, New Zealand; 4 Pacific Radiology Group, Palmerston North, New Zealand; University of Castilla-La Mancha, SPAIN

## Abstract

**Introduction:**

Contusion injuries are common in sport, but our knowledge of the responses to injury primarily come from animal studies and research using eccentric exercise. Therefore, the aim of this study was to develop a model of contusion injury in human participants and, additionally, investigate and compare physiological responses to four impact loads.

**Methods:**

Thirty-two males were exposed to a single impact of either 4.2, 5.2, 6.2 or 7.2kg, dropped from 67 cm, on to the *vastus lateralis* of one leg. Maximum voluntary and electrically induced quadriceps force, and pressure pain threshold were measured, and blood sampling carried out, prior to and 30min, 24, 48 and 72h post-impact. Magnetic resonance imaging was carried out 24h post-impact to quantify oedema.

**Results:**

Despite impact force with 7.2kg (1681.4 ± 235.6 N) not being different to 6.2kg (1690.7 ± 117.6 N), 7.2kg resulted in greater volume of oedema, voluntary force loss, pain and elevations in creatine kinase than the other loads. Although electrically induced force changed over time, post-hoc analysis failed to identify any changes. Interleukin-6 and prostaglandin-E_2_ did not change over time for any of the loads. Significant correlations were found between oedema volume, pressure pain threshold and maximum voluntary contraction force.

**Conclusions:**

This is the first experimental study to investigate traumatic loading of skeletal muscle and the subsequent physiological responses associated with contusion injuries in humans. The absence of immediate elevations in creatine kinase and changes in electrically induced force suggest impact, with forces similar to those experienced in contact sport, does not cause significant, direct damage to skeletal muscle. However, the relationship between oedema volume, changes in pressure pain threshold and maximum voluntary contraction force suggests central inhibition plays a role in contusion-related muscle dysfunction.

## Introduction

Classified as direct, non-penetrative muscle trauma, contusion injuries are common in sport [1] and as a result of falls and accidents in everyday life [2]. A contusion injury may occur when skeletal muscle is rapidly compressed between a solid external object and a bone [3], for example in contact team sport or combat sport when a knee or shoulder compresses the quadriceps against the femur. Depending on the severity of the injury, symptoms may include a loss of function, swelling, oedema/haematoma, localised muscle pain, decreased range of motion [1] and inflammation [4].

Our understanding of the symptoms, mechanisms and healing processes of contusion injury primarily come from animal models and, to a lesser extent, clinical studies in humans. Often done retrospectively, clinical studies, where an individual presents to a medical facility/clinician after injury [5, 6], are inherently problematic [2, 7] as the timing of injury, forces involved and influence of other behaviours before, during and after injury are unable to be controlled or fully accounted for. As such, the unique nature of each injury makes it difficult to develop an accurate understanding of the physiological responses to contusion injury and the efficacy of recovery modalities [8].

Therefore, in order to provide the experimental control needed to understand injury, the drop load/mass model in animals has been used extensively to investigate the histological, inflammatory and functional responses to injury, as well as various therapeutic modalities aimed at enhancing post-injury recovery (reviewed by de Souza & Gottfried [9]. Typically, this model uses a metallic mass dropped down a guide tube onto the hind limb of an anaesthetised rat or mouse, resulting in considerable force loss in the hours and days after injury [7, 10–12]. However, as muscle contraction is induced by maximal electrical nerve stimulation, it is difficult to directly relate these findings to humans [7] as the relationship between voluntary force production, pain and other factors cannot be ascertained.

Despite the numerous drop load models used in animal studies, there is no equivalent model for soft tissue injury in humans. Therefore, whether the relationships between impact forces and subsequent responses in animal studies can be extrapolated to humans remains unclear. Experimental models in humans have predominantly used eccentric exercise to bring about microstructural muscle damage, decreased function, delayed onset of muscle soreness (DOMS) and inflammation [13]. Although the resulting symptoms of eccentric exercise-induced muscle damage (EIMD) are similar to contusion and strain injury, the mechanism of injury and the repair processes are different to these other forms of muscle trauma [14, 15].

Recently, Naughton et al. [16] sought to simulate the collisions that occur in contact team sport by adapting the drop load model of Souza and Gottfried [9]. Exposing the anterior, lateral and posterior regions of the thighs to repeated impacts resulted in soreness and reductions in power and speed for 48 h post-impact. While the model of Naughton et al. [16] goes some way to replicating the outcomes of the repeated impacts that occur in contact team sports and combat sports, it is not representative of a single impact contusion injury.

Although insight into the mechanisms, responses and repair processes of contusion injury have been established using animal models, a similar model in humans would provide a means to investigate acute responses, chronic adaptation and modalities of recovery in a species specific, ecologically valid and controlled manner. Therefore, the primary objective of this study was to develop a model of contusion injury in human participants. In order to ensure that the new model causes a significant contusion injury, and is safe for future use, the following criteria had to be met: 1) moderate to severe oedema is observed with magnetic resonance imaging (MRI) 24 h post-impact [17], 2) pain at the site of impact is immediate and long lasting [18], 3) the magnitude and effect size of contusion related force loss is similar to that observed with animal contusion injury models and eccentric exercise models [19, 20] and 4) no complications, such as bone lesions, are evident. The secondary objective of this study was to investigate and compare the neuromuscular, pain, inflammatory and creatine kinase (CK) responses to four impact loads and the relationships between criterion measures and impact variables.

## Methods

### Participants

Thirty-two healthy males (height 179.7 ± 8.49 cm; body mass 87.65 ± 14.78 kg; age 25.8 ± 6.7 years) participated in this study. Participants were free of lower body injury, cardiovascular disease, anaemia and bleeding and clotting disorders (such as von Willebrand disease or haemophilia) and had all previously experienced sport-related contusion injury without complication. Participants were asked to refrain from physical exercise in the 48 h prior to and during the 72 h of the experimental trial. Additionally, over this same period, participants were instructed to abstain from alcohol and medications or supplements that may alter their response to impact. Participants were randomly allocated into four groups (4.2 kg, 5.2 kg, 6.2 kg and 7.2 kg) for the experimental trials.

At least one week before their trial, participants were familiarised with the criterion measures and maximum transcutaneous electrical stimulation (TES) output current was identified [21].

This study was approved by the Massey University Human Ethics Committee (13/64) and was conducted in accordance with the standards set out by the *Declaration of Helsinki*. All participants were informed of the aim of the study, the procedures involved and potential risks and benefits before providing written informed consent.

### Experimental protocol

For the experimental trial, participants presented to the laboratory and pre-impact blood sampling and criterion measures of pain (pressure pain threshold (PPT)), TES and maximal voluntary isometric contraction force (MVIC) were made, in that order. Depending on the group, one of four loads was then dropped from 67 cm onto the *vastus lateralis* of one leg. Blood sampling and criterion measures were repeated 30 min post-impact and at the same time of day 24, 48 and 72 h later. Additionally, MRI of the impact site was carried out 24 h (± 1 h) post-impact.

After data collection was completed on each group (starting with 4.2 kg), results were assessed to identify whether the criteria for the model of contusion injury had been met. If the following criteria were not met, then the load was increased for the next group: moderate to severe oedema observed with magnetic resonance imaging (MRI) 24 h post-impact; immediate and prolonged pain at the site of impact; voluntary force loss similar to that seen with animal contusion injury models and eccentric exercise models; and, importantly, no observed complications, such as bone lesions.

### Procedures and measurements

#### Impact

A 1 kg custom-made implement consisting of a spherical, hard resin head (10 cm in diameter) and metal tubular stem was used to bring about impact. Additional weight was loaded onto the stem and clamped in place so that loads of 4.2, 5.2, 6.2 and 7.2 kg were used during the trials; each load was applied to one group of 8 participants. Using a modified drop-load method [7], the loaded implement was dropped from a standardised height (67 cm) down a low friction polyvinyl chloride (PVC) tube onto the participants maximally contracted *vastus lateralis*. Pilot work showed that dropping the lightest load from this height resulted in mild oedema and therefore this height was used for the duration of the study. Although a greater contusion injury is likely to occur when a load is dropped on to a relaxed muscle [22], having participants maximally contract the quadriceps immediately prior to and during impact standardised the state of the muscle and reduced any effect that anticipation/ flinching may have had on impact force and subsequent responses. The centre of impact was half the distance between the superior boarder of the patella and the anterior superior iliac spine.

During the impact, participants were positioned on their side with their leg supported so that the thigh was horizontal, and the centre of impact was positioned perpendicular to the PVC tube.

Impact force was calculated using an accelerometer (X200-4, Gulf Coast Data Concepts, MS, USA), measuring at 800Hz. Accelerometer data was exported into Chart for Windows software, filtered and peak impact force, duration of the impact and impulse were calculated.

In order to estimate impact area (cm^2^), high viscosity black paint was applied to the hard resin head of the implement. Immediately after impact, the diameter of the paint mark at the site of impact was measured using callipers and the radius of the paint mark was used to calculate impact depth (cm), volume of tissue compressed (cm^3^) and average pressure (kPa) across the area of the penetrating surface. Assuming that there is uniform deformation of the tissue during impact, the penetration depth of the hard resin head (radius R) may be calculated as 2 *R* sin^2^(*r*/2*R*), where *r* is the radius of the black paint mark. On the same basis, the volume of compressed tissue may be calculated as 4 π *R*^3^/3 - π *h* (3*c*^*2*^ *+ h*^2^)/6 where *c* and *h* are equal to *R* sin (*r*/*R)* and 2 R cos^2^(r/2R), respectively.

#### Pressure-pain threshold

PPT was measured using a handheld digital algometer (Force Ten FDX, Wagner Instruments, Greenwich, USA). Participants were seated on a custom-made isometric dynamometer with the knee flexed and quadriceps relaxed. The rubber head of the algometer (area of 1cm^2^) was positioned perpendicular to the centre of impact and force was applied at a constant rate (5 N/s) until the participant verbally indicated that the force being applied was painful [23]. Measures were made twice, separated by 60 s, and the amount of pressure (kPa) associated with the perceived pain was recorded and the peak value used for analysis.

#### Maximal voluntary isometric contraction force

Participants were seated on a custom-made isometric dynamometer so that the hip and knee were at 90°, and the upper and lower back were supported. A seat belt was fastened around the hips and an inextensible strap was attached to the ankle, approximately 2 cm proximal to the medial malleolus. An S-beam load cell (Sensortronics, USA) was attached to the ankle strap and dynamometer, and MVIC force was recorded via a custom-made amplifier, data acquisition system (PowerLab, ADInstruments, Australia) and Chart for Windows v8.1 software (ADInstruments, Australia). Participants performed 2 x 5 s maximal isometric contractions of the quadriceps, with each effort separated by 60 s. The highest value was used for analysis.

#### Transcutaneous electrical stimulation force

Two 45 x 90 mm electrodes (Empi, Min, USA) were positioned longitudinally 5 cm either side (10 cm apart) of the centre of impact. Once attached, electrodes remained in place for pre-impact and 30 min post-impact measurements. To ensure consistent electrode placement for measurements at 24, 48 and 72 h post-impact, a permanent marker was used to trace around the outside of each electrode.

Using the maximal stimulation output current (569.4 ± 97.6 mA), identified during familiarisation, a doublet (400 V, 0.001 s pulse width with 0.01 s inter-pulse gap) was applied to the relaxed *vastus lateralis* via a constant current stimulator (Digitimer DS7, Digitimer Ltd, England) and force was recorded via the S-beam load cell, custom-made amplifier and Chart for Windows software. Two measures were made, separated by 60 s, with the maximal value used for analysis.

#### Magnetic resonance imaging

MRI was carried out on a 1.5T Aera MRI scanner (Siemens, Erlangen, Germany). Participants were positioned supine on the table with their knees supported for lumbar comfort. To identify the injured area, a vitamin E capsule was placed on the centre of impact and two-body matrix, receive-only coils were placed to provide lengthwise coverage of the abductors. The MRI protocol included DIXON fat-suppressed (FS) T2-weighted turbospin-echo (TSE) (repetition time (TR) 4750 ms; echo time (TE) 81 ms; field-of-view (FOV) 360 × 360 mm; slice thickness 5 mm; interslice gap 0 mm); 384 x 288 matrix, axial SPAIR FS T2-weighted TSE (TR 4160 ms; TE 61 ms; FOV 230 × 230 mm; slice thickness 4 mm; interslice gap 0 mm); 256 x 218 matrix, and sagittal T1 weighted TSE (FOV 240 × 240 mm; slice thickness 3 mm; interslice gap 1 mm); 384 x 269 matrix.

Oedema was classified as none, minimal, mild, moderate or severe by a musculoskeletal imaging specialist (Pacific Radiology, Palmerston North, New Zealand) and a numerical value was allocated to each category for analysis: none = 0, minimal = 1, mild = 2, moderate = 3, severe = 4.

**Fig 1:**
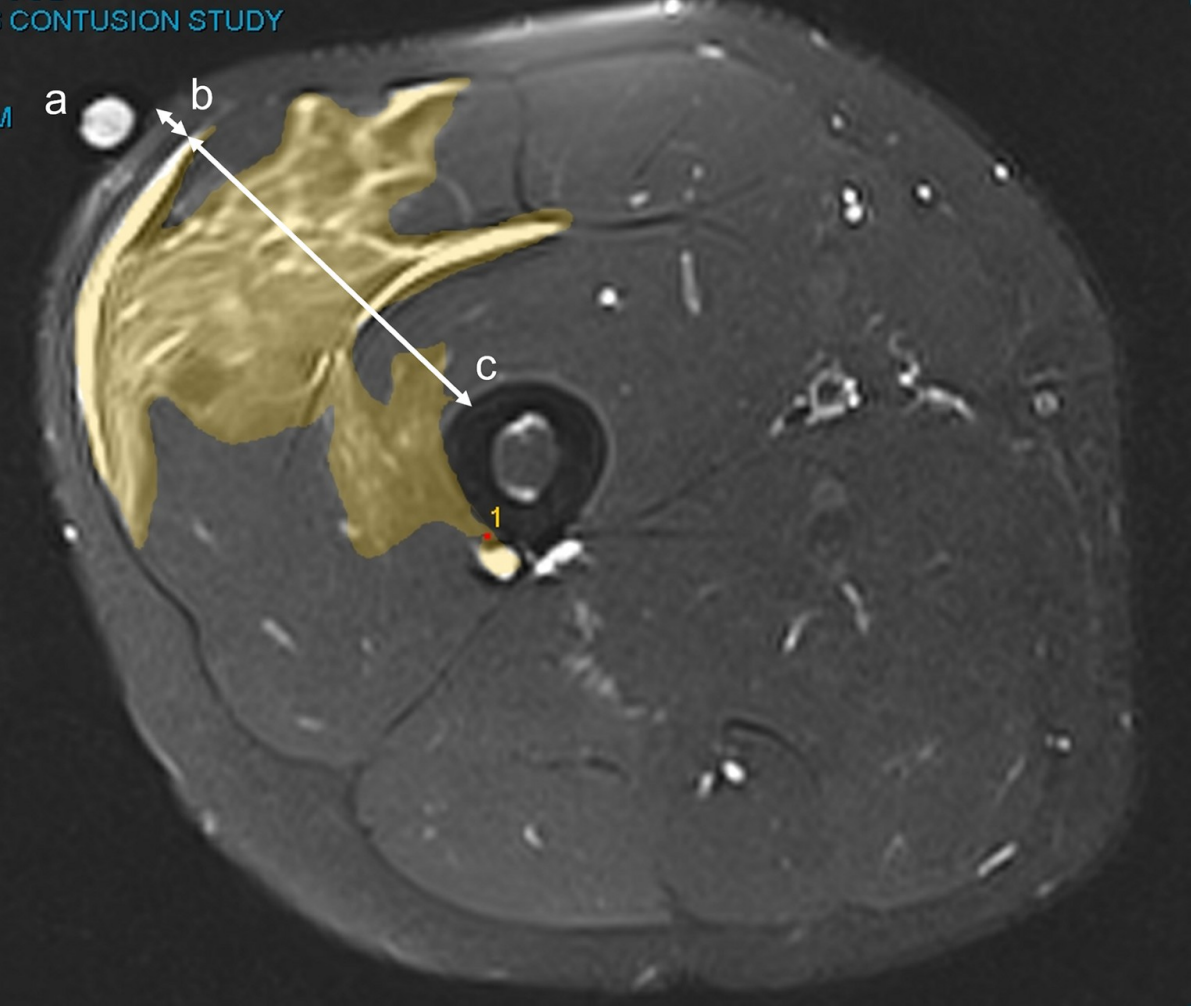
MRI image, at 24 h post-impact, of oedema in the quadriceps after impact with 7.2 kg. Oedema is identified as regions of high intensity and area measured (highlighted area) so that oedema volume could be quantified. Additionally, fat (b) and muscle thickness (c) were measured at the site of impact. (a) denotes the vitamin E capsule marker placed on the centre of impact. Axial T2-weighted TSE SPAIR FS (TR 4160 ms; TE 61 ms; FOV 230 × 230 mm; slice thickness 4 mm; interslice gap 0 mm) was used for imaging.

Additionally, the volume of oedema was identified by calculating the area of hyperintensity on each axial slice and multiplying this area by the number of slices where oedema was visible and by the depth of each slice (4 mm). Subcutaneous fat and muscle tissue thickness was calculated along a line placed perpendicular to the femur and surface of the impact site (Fig 1).

#### Blood sampling and analysis

Venous blood samples were collected from the antecubital vein of one arm into 9 ml K_3_EDTA vacutainer tubes (Beckton Dickinson, UK). Samples were centrifuged at 4 ˚C at 1500 rpm for 10 min, plasma was removed, and aliquots stored at -80 ˚C until analysis.

CK activity was determined using a Vitalab Flexor clinical chemistry analyser (Vital Scientific NV, Netherlands) and a Roche CK-NAC liquid assay kit (Roche Diagnostics GmbH, Mannheim, Germany). Interleukin-6 (IL-6) was determined using a high sensitivity ELISA kit (cat. no. DY206; R&D Systems, Minneapolis, Minn). Prostaglandin-E_2_ (PGE_2_) was determined by ELISA (cat. No KHL1701HU, Invitrogen, Vienna, Austria).

### Statistical analysis

Two-factor mixed analysis of variance (ANOVA) was used to identify changes over time and the time x load interaction for PPT, MVIC, TES and blood variables. One-way ANOVA was performed to investigate differences between loads for physical characteristics, impact-related variables and MRI volume. Where a main effect or an interaction was identified, post-hoc analysis with Bonferroni adjustment was carried out.

Data were tested for normality using the Shapiro-Wilk test. CK, PGE_2_ and IL-6 were not normally distributed and, therefore, data were log transformed prior to analysis.

Mauchley’s test was used to assess sphericity (ε) and, where the assumption of sphericity was violated, adjustments to the degrees of freedom were made (ε > 0.75 = Huynh–Feldt; ε < 0.75 = Greenhouse–Geisser).

Pearson’s correlation coefficient (*r*) was calculated using the percent change, from pre-impact, of pooled data (*n* = 32) to investigate the relationships between changes in PPT, TES, MVIC, oedema, impact force and impulse at all post-impact timepoints. Correlation coefficients were classified as positively or negatively very weak (*r* < 0.19, or -0.19), weak (*r* = 0.20 to 0.39, or -0.20 to -0.39), moderate (*r* = 0.40 to 0.59, or– 0.40 to -0.59), strong (*r* = 0.60 to 0.79, or -0.60 to -0.79) and very strong (*r* = 0.80 to 1.0, or -0.80 to -1.0) [24]. Effect sizes (*d*) and 95% confidence intervals (CI) were calculated as change from baseline and classified as small (0.2 to 0.49), medium (0.5 to 0.79) and large (≥0.80) [25].

To identify reliability of measures for PPT, MVIC and TES, intraclass correlation coefficient (ICC) and CI were calculated using a two-factor mixed-effect model with absolute agreement. Maximum baseline values from 12 participants were compared to values obtained on a separate occasion, at least 5 days prior to the experimental trail. Reliability was classified as poor (ICC < 0.5), moderate (0.5 to 0.75), good (0.75 to 0.9) and excellent (> 0.9) [26]. All analysis was carried out using IBM SPSS Statistics v23 (IMB Corp. NY, USA). Statistical significance was set at *P* < 0.05, and all data are presented as mean ± standard deviation to two significant places.

## Results

### Participant characteristics

Participant characteristics were not different between groups (height, *P* = 0.592; weight; *P* = 0.644, age; *P =* 0.071, MRI derived subcutaneous fat; *P* = 0.389, and muscle thickness at impact site, *P* = 0.991, Table 1).

**Table 1:** Participant characteristics by load.

	Load			
	4.2 kg (*n* = 8)	5.2 kg (*n* = 8)	6.2 kg (*n* = 8)	7.2 kg (*n* = 8)
Age (years)	28.6 ± 7.2	24.7 ± 5.2	21.1 ± 2.8	28.6 ± 8.5
Height (cm)	178.1 ± 8.0	182.0 ± 8.3	181.3 ± 8.5	176.9 ± 9.7
Body mass (kg)	89.6 ± 15.4	91.6 ± 16.3	87.0 ± 9.9	82.4 ± 17.6
Subcutaneous fat thickness (cm)	0.58 ± 0.22	0.77 ± 0.50	0.52 ± 0.34	0.52 ± 0.08
Muscle thickness (cm)	5.91 ± 0.57	5.92 ± 1.27	6.01 ± 0.48	5.89 ± 0.67

Data are mean ± SD.

### Reliability of measurements

Excellent reliability was found for all criterion measures (PPT = ICC, 0.97 (95% CI, 0.9–0.99); MVIC = 0.93 (0.78–0.98); TES = 0.99 (0.98–0.99)).

### Impact variables

Impact force differed between loads (*P* < 0.001) with 4.2 kg lower than 5.2 kg (*P* = 0.023), 6.2 kg (*P* < 0.001) and 7.2 kg (*P* < 0.001). Additionally, 5.2 kg was lower than 6.2 kg (*P* = 0.014) and 7.2 kg (*P* = 0.020). The greatest impact force occurred with 6.2 kg (1690.65 ± 117.58 N), however this was not significantly different to that for 7.2 kg (1681.36 ± 235.60 N).

Impulse was significantly different between loads (*P* < 0.001) with the highest impulse occurring with 7.2 kg (113.25 ± 24.42 N/s); this was different to all other loads. Similarly, impact duration differed between loads (*P* < 0.001). Impact duration for 4.2 kg was different to 7.2 kg (*P* < 0.001) and 5.2 kg was different to 7.2 kg (*P* = 0.002); no other differences between loads were found. The greatest impact duration occurred with 7.2 kg; however, this was not different to 6.2 kg (*P* = 0.064). The volume of tissue compressed (*P* = 0.604) and depth of penetration (*P* = 0.605) did not differ between loads. Average impact pressure differed between loads (*P* = 0.002) with 4.2 kg significantly lower than 6.2 kg (*P* = 0.002) and 7.2 kg (*P* = 0.007). No other differences between loads were found. Results for all impact variables are outlined in Table 2.

**Table 2:** Impact variables associated with dropping one of four loads onto the centre of the contracted *vastus lateralis*.

	Load			
	4.2kg (*n* = 8)	5.2 kg (*n* = 8)	6.2 kg (*n* = 8)	7.2 kg (*n* = 8)
Impact force (N)	1221.86 ± 90.40	1449.07 ± 77.46 ^a^	1690.65 ± 117.58 ^a^^b^	1681.37 ± 235.60 ^a^^b^
Impact duration (s)	0.04 ± 0.01	0.05 ± 0.01	0.05 ± 0.01^a^	0.07 ± 0.01^a^^b^
Impulse (N/s)	51.69 ± 11.29	73.37 ± 12.49	91.76 ± 15.66^a^^b^	113.24 ± 24.42 ^a^^b^^c^
Average impact pressure (kPa)	52.96 ± 4.57	68.75 ± 10.69	79.35 ± 10.67 ^a^	76.50 ± 20.92 ^a^
Compressed tissue (cm^3^)	41.58 ± 3.11	37.07 ± 9.45	37.21 ± 8.5	40.84 ± 10.77
Impact depth (cm)	1.73 ± 0.07	1.61 ± 0.24	1.62 ± 0.20	1.70 ± 0.25

Data are mean ± SD.

^a^ different to 4.2 kg

^b^ different to 5.2 kg

^c^ different to 6.2 kg.

### PPT

The amount of pressure required to produce a sensation of pain was reduced over time (*P* < 0.001; Table 3) and a time x load interaction was observed (*P* < 0.001). Impact with 6.2 kg and 7.2 kg resulted in early and prolonged pain, whereas significant changes in PPT for 4.2 kg and 5.2 kg were first observed 24 h post-impact and were not significantly different to pre-impact values at 72 h. Medium and large effect sizes were found at all post-impact time points, with the greatest effect sizes found at 24 h (*d* = 2.3) and 48 h (*d* = 2.0) for 7.2 kg.

**Table 3:** Changes in pressure pain threshold (PPT), maximal voluntary isometric contraction force (MVIC) and transcutaneous electrical stimulation force (TES) prior to and up to 72 h post-impact with four different loads.

	Time				
PPT (kPa)	Pre-Impact	30 min post	24 h post	48 h post	72 h post
4.2 kg	854±185	704±194 (-0.8, -1.8–0.2)	621±186 ^a^ (-1.2, -2.3- -0.1)	741±198^c^ (-0.6, -1.6–0.4)	726±167 (-0.7, -1.7–0.3)
5.2 kg	1051±182	912±281 (-0.5, -1.5–0.5)	770±254 ^a^^b^ (-1.2, -2.2- -0.1)	766±294 ^a^ (-1.1, -2.1- -0.03)	897±371 (-0.5, -1.4–0.5)
6.2 kg	1225±333	809±313 ^A^ (-1.3, -2.4- -0.2)	681±350 ^A^^b^ (-1.6, -2.7- -0.5)	797±275 ^A^^c^ (-1.4. -2.5- -0.3)	955±344 ^a^^C^^d^ (-0.8, -1.8–0.2)
7.2 kg	1193±252	683±349 ^A^ (-1.7, -2.8- -0.5)	550±298 ^A^^b^ (-2.3, -3.6- -1.1)	643±269 ^A^ (-2.0, -3.2- -0.8)	788±349 ^a^^c^^d^ (-1.3, -2.4- -0.2)
MVIC (N)					
4.2 kg	665±171	614±158 (-0.3, -1.2–0.7)	623±147 (-0.3, -1.2–0.7)	634±140 (-0.2, -1.2–0.8)	659±140 (-0.03, -1.0–0.9)
5.2 kg	691±93	621±114 ^a^ (-0.7, -1.7–0.3)	630±139 (-0.5, -1.5–0.5)	640±151 (-0.4, -1.4–0.6)	661±131 (-0.3, -1.3–0.7)
6.2 kg	703±82	643±67 ^a^ (-0.8, -1.8–0.2)	648±100 (-0.6, -1.6–0.4)	680±69 (-0.3, -1.3–0.7)	685±62 (-0.3, -1.2–0.7)
7.2 kg	658±108	508±149 ^A^ (-1.1, -2.2- -0.1)	522±170 ^A^ (-1.0, -1.9–0.1)	556±139 ^a^ (-0.8, -1.8–0.2)	564±83 ^A^ (-1.0, -2.0–0.1)
TES (N)					
4.2 kg	274±32	271±30 (-0.1, -1.08–0.9)	267±36 (-0.2, -1.14–0.8)	251±51 (-0.6, -1.6–0.4)	261±31 (-0.4, -1.4–0.5)
5.2 kg	283±63	279±66 (-0.06, -1.0–0.9)	272±59 (-0.2, -1.2–0.8)	275±64 (-0.1, -1.1–0.9)	263±70 (-0.3, -1.3–0.7)
6.2 kg	305±36	295±28 (-0.3, -1.3–0.7)	305±46 (0.01, -1.0–1.0)	300±36 (-0.1, -1.1–0.8)	303±40 (-0.04, -1.0–0.9)
7.2 kg	284±42	289±43 (-0.1, -0.9–1.1)	275±33 (-0.3, -1.2–0.7)	268±32 (-0.4, -1.4–0.5)	273±30 (-0.3, -1.3–0.7)

Data are mean ± SD (Cohens *d*, 95% CI).

^a^ different to pre-impact, *P* < 0.05

^A^ different to pre-impact, *P* < 0.0005

^b^ different to 30 min post-impact, *P* < 0.05

^c^ different to 24 h post-impact, *P* < 0.05

^C^ different to pre-impact, *P* < 0.0005

^d^ different to 48 h post-impact, *P* < 0.05.

### MVIC

MVIC force was reduced over time (*P* < 0.001, Table 3). However, a time x group interaction was not observed (*P* = 0.145). Large effect sizes were found at 30 min post-impact for 6.2 kg (*d* = - 0.80) and all post-impact times points for 7.2 kg (all *d* < - 1.3). All other effect sizes were small and medium.

### TES

The force produced by electrical stimulation decreased over time (*P* = 0.002; Table 3). However, post-hoc analysis failed to identify any significant changes between time points for any of the loads and no interaction was found (*P* = 0.372). Except for 4.2 kg at 48 h (*d* = -0.55), small effect sizes (all *d* < 0.49) were found across all time points and for all loads.

**Fig 2:**
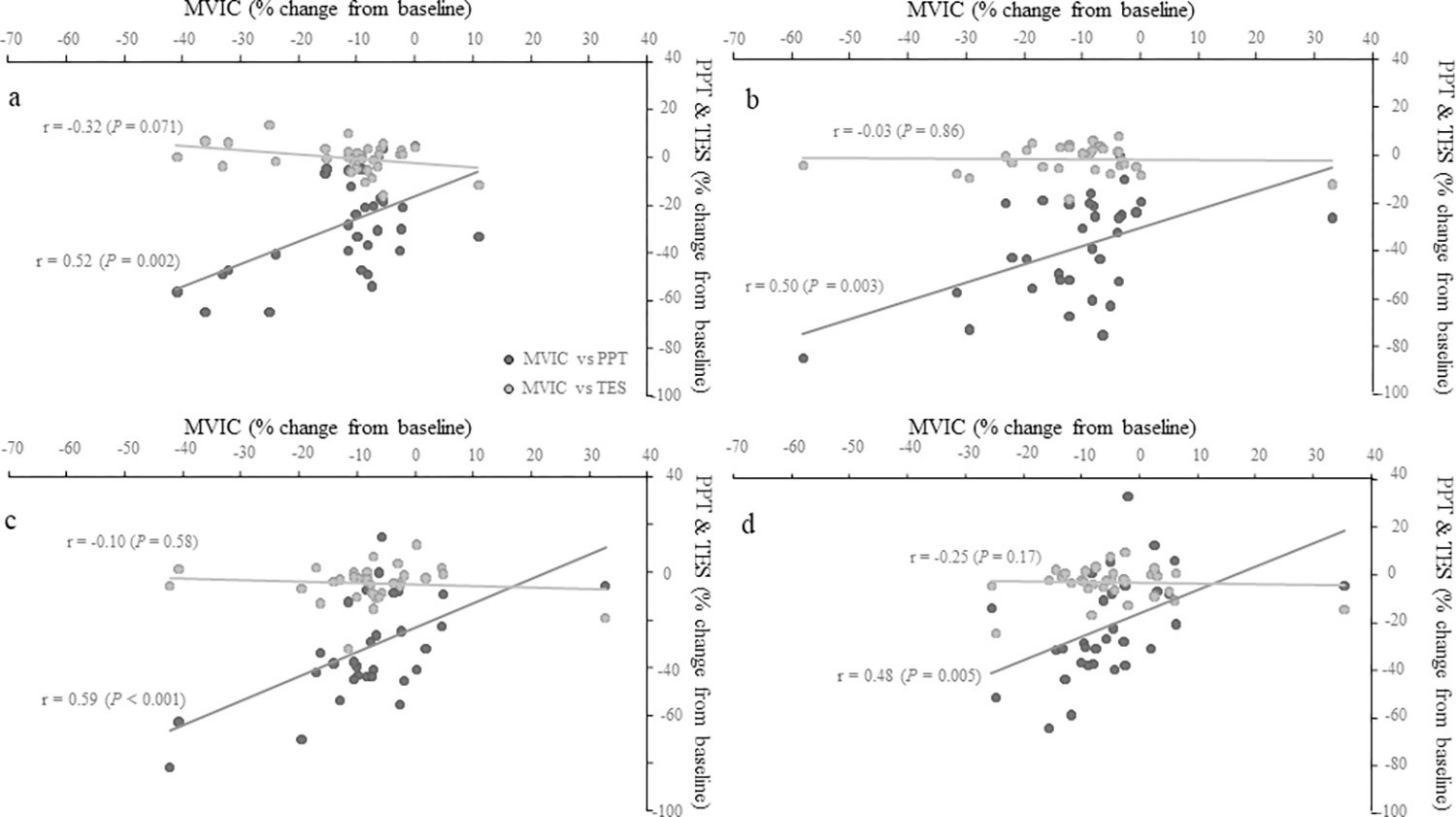
Correlations (Pearson’s *r*) between percent change (from pre-impact) in MVIC and PPT (black circles), and between MVIC and TES (grey circles) at 30 min (a), 24 h (b), 48 h (c) and 72 h (d) post-impact. *n* = 32.

When data were pooled (*n* = 32), significant, moderate correlations were found between changes in PPT and MVIC across all post-impact time points. In addition to the time point specific correlations shown in Fig 2, changes in PPT at 30 min, 24 h, 48 h and 72 h post-impact were all correlated to changes in MVIC 30 min, 24 h, 48 h and 72 h post-impact (all *r* = 0.40–0.48, *P* < 0.050). Conversely, correlations between changes in TES and MVIC were weak or very weak and non-significant (all *P* > 0.070).

Impulse was significantly related to changes in PPT 30 min post-impact (*r* = -0.35, *P =* 0.050) and MVIC 72 h post-impact (*r* = -0.46, *P =* 0.008). Impact time was weakly correlated with changes in PPT 30 min post-impact (*r* = -0.35, *P =* 0.043) and changes in MVIC at 30 min (r = -0.39, *P* = 0.026) and 72 h post-impact (*r* = -0.39, *P =* 0.027). No other significant correlations were found between MVIC, PPT, TES and impulse, impact time and impact force.

### MRI

**Fig 3:**
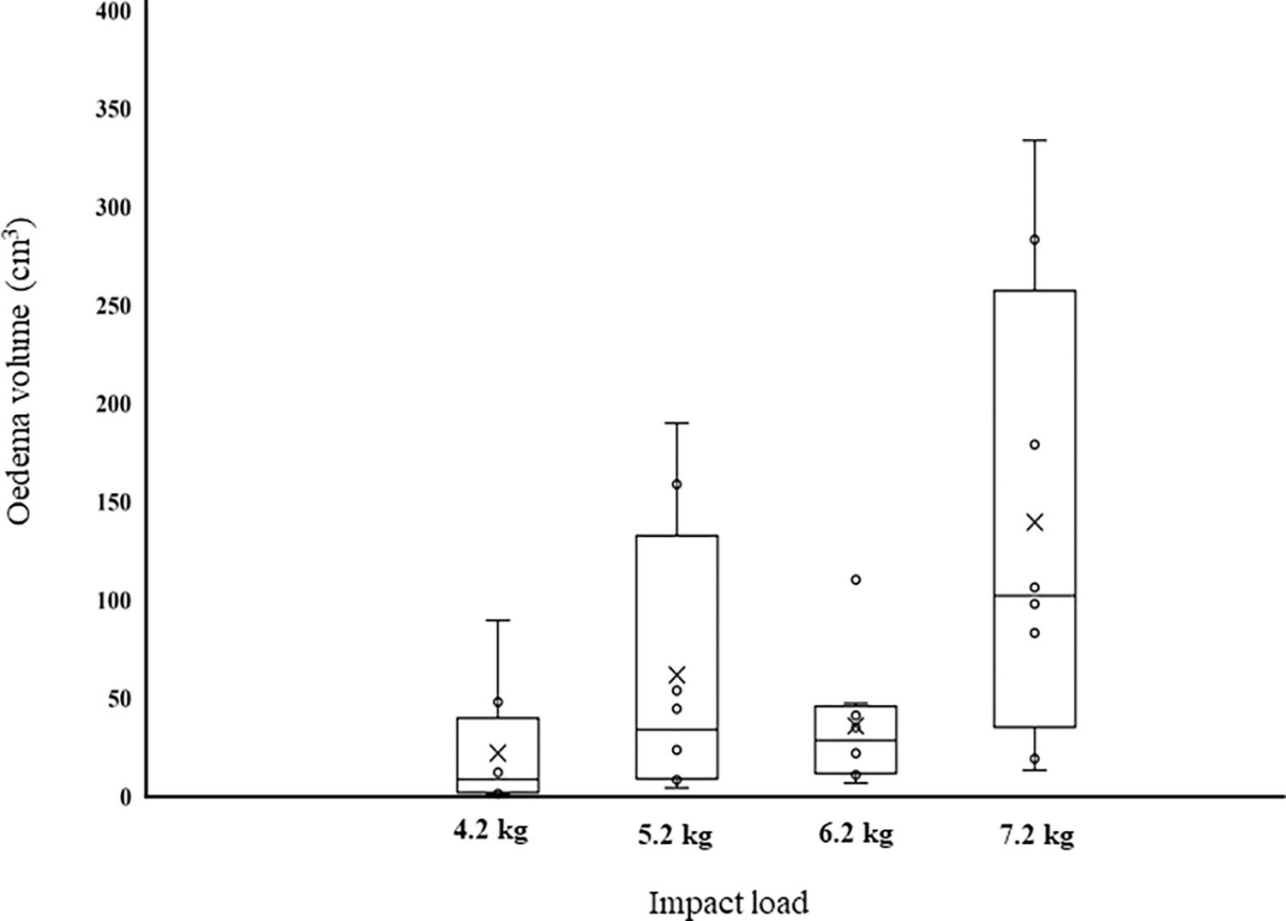
Box plot of individual and group distribution of oedema volume, measured by MRI 24 h post-impact with four different loads (*n* = 8 per group).

The number of participants exhibiting moderate oedema increased as the load increased (4.2 kg, *n* = 1; 5.2 kg, *n* = 2; 6.2 kg, *n* = 4; 7.2 kg, *n* = 6). Although the severity of oedema increased across loads (4.2 kg = 0.86 ± 0.64 (minimal to mild); 5.2 kg = 1.0 ± 0.75 (minimal to mild), 6.2 kg = 1.38 ± 0.74 (mild to moderate), 7.2 kg = 1.75 ± 0.46 (mild to moderate)), no significant difference between loads was found (*P* = 0.055). However, the volume of oedema differed between loads (*P* = 0.016) with the greatest oedema occurring with 7.2 kg (139.30 ± 117.40 cm^3^). This was greater than 4.2 kg (21.81 ± 31.36 cm^3^, *P* = 0.019) and 6.2 kg (35.73 ± 33.45 cm^3^, *P* = 0.048), but not 5.2 kg (61.53 ± 72.18 cm^3^, *P* = 0.246). Large individual variations in oedema were observed (Fig 3), however these were not explained by differences in tissue thickness (*r* = -0.12), impact (*r* = - 0.17, *P =* 0.36) or impulse (*r* = 0.34, *P =* 0.055). However, oedema was correlated to impact duration (*r* = 0.47, *P =* 0.007).

When data from all loads were pooled (*n* = 32), oedema volume was negatively related to the decrease in pressure required to produce a sensation of pain (PPT), with moderate, significant correlations found at 30 min (*r* = -0.58, *P* < 0.001), 24 h (*r* = -0.61, *P* < 0.001), 48 h (*r* = -0.524, *P* = 0.002) and 72 h (*r* = -0.47, *P* = 0.007) post-impact. Similarly, reductions in MVIC were strongly (30 min post-impact, *r* = -0.81, *P* < 0.001) and moderately (24 h *r* = -0.54, *P* = 0.002; 48 h *r* = -0.56, *P* = 0.002; 72 h *r* = -0.40, *P* = 0.022) correlated to oedema volume. Correlations between oedema and changes in TES were not significant (all *P* > 0.1). A relationship was found between oedema volume and impact time (*r* = 0.47, *P =* 0.007); oedema was not related to other impact related variables.

### Blood variables

None of the measured blood variables changed over time (CK, *P* = 0.213; IL-6, *P* = 0.145, PGE_2_, *P* = 0.921; Table 4). However, a time x load interaction was found for CK (*P* < 0.001); CK was significantly greater than pre-impact at 24 h, 48 h and 72 h post-impact with 7.2 kg, only. No other interactions were found (IL-6, *P* = 0.378; PGE_2_, *P* = 0.582). Large effect sizes were found at 24 h and 48 h for CK and at 72 h for PGE_2_ with 7.2 kg; however, this represents a decrease in PGE_2_ from baseline so is unrelated to the impact response. All other effect sizes were small or medium.

**Table 4:** Creatine kinase (CK), interleukin-6 (IL-6) and prostaglandin-E_2_ (PGE_2_) prior to and up to 72 h after impact with four different loads.

	Time				
CK (U/L)	Pre-Impact	30 min post	24 h post	48 h post	72 h post
4.2 kg	332±269	331±250 (-0.003, -0.9–1.0)	285±161 (-0.2, -1.2–0.8)	223±119 (-0.5, -1.5–0.5)	186±74 (-0.7, -1.8–0.3)
5.2 kg	242±128	222±129 (-0.2, -1.1–0.8)	226±104 (-0.1, -1.1–0.8)	171±64 (-0.7, -1.7–0.3)	193±104 (-0.4, -1.4–0.6)
6.2 kg	453±334	408±285 (-0.1, -1.2–0.8)	518±416 (0.2, -0.8–1.2)	516±522 (0.1, -0.8–1.1)	423±410 (-0.1, -1.1–0.9)
7.2 kg	176±58	175±59 (-0.03, -1.0–0.9)	327±163^a^^,^^b^ (1.2, 0.2–2.3)	530±603 ^a^^,^^b^ (0.8, -0.2–1.8)	887±1524 ^a^^,^^b^ (0.7, -0.3–1.7)
IL-6 (pg/mL)					
4.2 kg	38±56	37±53 (-0.03, -1.0–1.0)	28±39 (-0.2, -1.2–0.8)	33±42 (-0.1, -1.1–0.9)	35±45 (-0.1, 1.0–0.9)
5.2 kg	26±23	26±24 (-0.02, -1.0–1.0)	25±23 (-0.1, -1.0–0.9)	17±10 (-0.5, -1.5–0.5)	34±26 (0.3, -0.7–1.3)
6.2 kg	36±24	37±24 (0.01, -1.0–1.0)	36±27 (-0.01, -1.0–1.0)	38±25 (0.1, -0.9–1.0)	40±28 (0.1, -0.8, 1.1)
7.2 kg	75±102	69±88 (-0.1, -1.0, 0.9)	73±97 (-0.02, -1.0, 1.0)	69±88 (-0.07, 1.0–0.9)	68±91 (-0.07, -1.0–0.9)
PGE_2_ (pg/mL)					
4.2 kg	113±49	-	99±36 (0.5, -1.3–0.65)	112±41 (-0.01, -1.0–1.0)	138±70 (0.4, -0.6–1.4)
5.2 kg	88±40	-	94±59 (0.1, -0.9–1.1)	104±41 (0.4, -0.6–1.4)	101±40 (0.3, -0.7–1.3)
6.2 kg	205±130	-	218±129 (0.1, -0.9–1.1)	172±74 (-0.3, -1.3–0.7)	169±119 (-0.3, -1.3–0.7)
7.2 kg	308±99	-	266±75 (-0.5, -1.5–0.5)	290±106 (-0.2, -1.2–0.8)	238±47 (-0.9, -1.9–0.1)

Data are mean ± SD (Cohen’s *d*, 95% CI).

^a^ different to pre-impact, *P* < 0.005

^b^ different to 30 min post-impact, *P* < 0.005.

Changes in blood variables were not correlated to impact variables. However, oedema volume correlated to changes in CK at 24 h (*r* = 0.70, *P* < 0.001) and 48 h (*r* = 0.49, *P* = 0.005), and changes in IL-6 at 24 h (*r* = 0.39, *P* = 0.026) post-impact. Changes in CK at 24 h (*r* = -0.63, *P* < 0.001), 48 h (*r* = -0.51, *P* = 0.001) and 72 h (*r* = -0.38, *P* = 0.031) were correlated to decreases in MVIC 30 min post-impact.

## Discussion

The primary aim of this study was to develop a valid model of contusion injury in human participants, as assessed by the presence of immediate and prolonged muscle pain, weakness and oedema. Additionally, this was the first study to investigate the physiological responses to several impact loads and identify the relationships between these responses and impact-related variables. All impact loads resulted in varying degrees of oedema and, therefore, contusion injuries of varying severity. Although no significant difference in oedema was observed between 5.2 and 7.2 kg, when considering the level of oedema in combination with changes in measures of pain and voluntary force production, 7.2 kg resulted in the most severe contusion injury and met all of our criteria for the development of a contusion injury model. While it seems intuitive that the greatest responses will occur with the heaviest impact load, impact force and impact pressure did not differ between 6.2 and 7.2 kg, and no relationships between impact force and changes in criterion measures were found. The relationships between volume of oedema, pain and voluntary force suggests that oedema influences both how a muscle feels and performs in the days after injury.

Due to the difficulties in researching contusion injuries in human participants [2, 4, 7], very little is known about the magnitude of force loss associated with injury. Fabiś [27] reported a strength deficit of 19.5 ± 3.4%, compared to the peak torque of the uninjured leg, in 4 patients up to 47 months after contusion injury. While these findings suggest that muscle function may remain compromised for an extended period, no acute measures were made. It is therefore unclear whether the changes in MVIC reported in our study represent the range of normal, expected levels of acute dysfunction. Drop load studies in rats have reported similar, or slightly greater, losses in force [7, 10–12] to the 23.7 and 21.9% decreases observed at 30 min and 24 h post-impact, respectively, with 7.2 kg. However, it is difficult to extrapolate the findings of animal studies to human participants due to differences in the volume of tissue injured, impact forces used, and the reliance on involuntary muscle force assessment in animal models.

While the impact forces occurring in animal studies result in considerable disruption to muscle fibres, intramuscular haematoma and inflammation [9], fractures to the underlying bone have been reported [7, 10, 11, 28]. Such severe injury may not be surprising given the dropped load typically equates to between 50 and > 100% of the animal’s body weight [10, 22, 28, 29]; in comparison, the loads used in our study range from 4.7 to 8.7% bodyweight. Additionally, and as an example of why the forces used in animal studies cannot be extrapolated to human contusion injuries, the impact forces occurring in animal studies may be as high as 1216 N per kg bodyweight [11], compared to a maximum of 9.4 N/kg bodyweight in this study. As such, it appears that the contusion injuries and subsequent responses occurring in animal studies are likely to be significantly more severe than, and not necessarily representative of, contusion injuries occurring in sport.

The impact forces in this study are similar to, or slightly lower than, impacts reported in contact sports. Impact forces reported in rugby union tackles are typically ~ 1600–2000 N [30, 31] and, likewise, impact forces from martial arts kicks range from ~1500–2000 N [32, 33]. These similar forces suggest that the drop load model developed here may be representative of the impacts in contact sport. As such, we may speculate that the responses are also representative of those resulting from sport-related contusion injury.

Despite similar impact forces for 6.2 and 7.2 kg, and a lack of difference between loads in the volume of tissue compressed and depth of impact, 7.2 kg produced a greater overall response. Impulse and impact time were related to PPT and MVIC 30 min post-impact and oedema volume, suggesting that these impact-related variables may influence the severity of injury. However, as many correlations were weak and not consistent across all time points, it is difficult to say definitively why a greater response was seen with 7.2 kg.

Although a difference in TES occurred over time, subsequent post-hoc analysis failed to identify any significant changes and, other than at one time point, only small effect sizes were found. Interestingly, changes in TES were not correlated to decreases in MVIC, suggesting that the functional components of the muscle were minimally damaged by impact. Given the relatively shallow depth of penetration, volume of compressed tissue and spherical shape of the implement used, it is unlikely that a significant amount of the *vastus lateralis* was directly compressed against the femur and certainly not enough to reduce whole quadriceps function (MVIC) by up to 23.7%, as occurred with 7.2 kg. Along with the lack of change in TES, the absence of immediate elevations in CK further suggests that even the highest impact forces failed to bring about significant, direct damage to skeletal muscle. Indeed, the findings of this study support the theory that contusion injury in humans may not necessarily be associated with significant structural damage to muscle tissue [1].

Elevations in CK, with 7.2 kg, suggest that changes to sarcolemmal permeability occurred to some extent. However, as with EIMD, elevations in CK were not observed immediately after impact [34] but continued to increase for 72 h post-impact (and possibly beyond). This delayed response is likely due to inflammation-related secondary damage caused by neutrophil and macrophage activity [35]. As well as allowing CK to enter circulation, damage to the sarcolemma also allows fluid to enter the muscle. Relationships between CK and T2 weighted MRI intensities (oedema) have been observed with EIMD [36, 37] and the same appears to be true of contusion injuries, with relationships found between oedema volume and CK 24 and 48 h post-impact. As MRI was only performed 24 h post-impact, it is unclear whether the relationship between CK and oedema volume is continued at each timepoint beyond 24 h.

Changes in CK at 24, 48 and 72 h were only related to force loss observed 30 min post-impact suggesting that initial force loss may be an indicator for the level of secondary damage, and associated release of CK and oedema, in the days after injury. Although Nosaka et al. [38] reported that initial force loss, after eccentric exercise, does not correlate well with subsequent changes in markers of EIMD, it appears that the responses to contusion injury are different. As previously shown with EIMD [39], our results confirm that CK is not a useful or valid proxy for changes in muscle function after contusion injury.

Despite an apparent lack of significant structural damage to the muscle, impact with 7.2 kg reduced voluntary force for the entire quadriceps group by up to 23.7%. This reduction may be, at least partly, explained by the correlations between oedema, pain and MVIC. Typically peaking at 24–48 h post impact, oedema causes swelling and increased pressure in and around the site of injury. This swelling may compress muscle fibres and lead to sensations of pain [1]. This effect may be magnified by an increase in pressure caused by contraction of the affected muscle, which in turn may limit voluntary force production [40]. Our results are similar to those of Graven-Nielsen et al. [41] who found that experimental pain in humans causes a decrease in MVIC, without a change in TES, suggesting that centrally-mediated inhibition contributes to the contusion-related force loss seen here.

Our findings support the role of oedema in contusion-related pain and show that, as the volume of oedema increases, the site of injury becomes more sensitive and painful. As inflammatory substances also peak at a similar time as oedema, we cannot discount the role of algesic substances, such as bradykinin and PGE_2_, which can act independently on nociceptors or increase the sensitivity of type IV receptors to oedema-related and externally-applied pressure [42]. Although circulating levels of PGE_2_ did not change across time for any of the loads, this may be due to the rapid metabolism of circulating PGE_2_ [43] and does not necessarily mean that PGE_2_ levels in the muscle were not elevated and contributing to the changes in PPT.

The time course of post-impact pain differed between the two lightest and two heaviest loads, with early and prolonged pain seen with 6.2 and 7.2 kg. The time course for the two heaviest loads is similar to that reported after repeated impacts to the thighs [16], while the pain observed with the lighter loads is akin to the delayed onset of muscle soreness that occurs with EIMD [44]. Acting as a conditioning stimulus, impact with the heavier loads may have sensitised high-threshold mechanosensitive (HTM) receptors to subsequent pressure [42], applied during the PPT test, resulting in sensations of localised pain 30 min post-impact. The lower loads, however, may not have provided sufficient stimulus to sensitise the HTM receptors, or sensitisation was short-lived, resulting in a lack of significant change in PPT at this time point. The various levels of pain experienced with all loads at 24, 48 and 72 h may be related to oedema and the influence of algesic substances, as previously mentioned.

IL-6 plays a role in muscle healing and regeneration after injury [45] and therefore we may have expected to see concentrations increase in response to impact. However, despite IL-6 concentrations being elevated in rat muscle after contusion injury [46] and in circulation after eccentric exercise [47], we failed to see a change. The reason for this finding is unclear. However, the relatively small amount of tissue injured by impact, particularly in comparison to animal models, and/or the absence of exercise, which can increase circulating IL-6 concentrations [48], may contribute to the lack of change.

As this is the first study to develop a model of soft tissue injury in humans, there are several limitations that warrant consideration and future investigation. Firstly, impact was done in isolation, whereas sport-related injury is likely to occur during exercise. Changes in blood flow and other acute responses to exercise may change the level of oedema and the inflammatory response, which may in turn alter the levels of pain and force loss presented here. Secondly, to get a greater understanding of the relationships between oedema, pain and MVIC, MRI should be assessed at all timepoints. As this is the first study of its kind, we decided to use only young, health male participants, who had previously experienced soft tissue injury without complication. However, given changes in the quality of muscle that occur with aging [49] and sex differences in pain perception [50], future research could investigate whether similar responses occur in females and older adults.

In conclusion, all impact loads used in this study resulted in contusion injuries of varying severity. However, impact with 7.2 kg met our criteria for the development of a contusion injury model. When dropped from 67 cm, this load caused immediate and long-lasting pain, significant reductions in voluntary force production and moderate levels of oedema in the days following impact. The responses to this load are similar to those reported in animal studies, EIMD and symptoms of sport-related contusion injury, and therefore this model is an ecologically valid representation of the contusion injuries that occur in sport. As there were no reported or observed complications, this load may be used in future studies to investigate the acute responses and chronic adaptations to contusion injury, as well as identifying methods to alleviate symptoms and expedite recovery. Our findings share a number of similarities with EIMD. However, the timelines of pain, relationships between CK and muscle function and lack of a measurable inflammatory response show that there are some differences between the two forms of muscle insult. Although there is no consensus on how to best treat contusion injuries, the relationships between oedema, PPT and MVIC support recent advice to focus on reducing oedema, using compression and elevation [51].
